# Engineering Performance of Expansive Soil Stabilized with Cement and Montmorillonite Adsorption Modifier

**DOI:** 10.3390/ma19122522

**Published:** 2026-06-11

**Authors:** Aiping Chen, Yong Cao, Wei Qi, Lihong Shu, Feiyang Liu, Ge Yang, Jianbiao Du, Tengfei Wang

**Affiliations:** 1 Sichuan Provincial Engineering Research Center of Rail Transit Lines Smart Operation and Maintenance, Chengdu Vocational & Technical College of Industry, Chengdu 610213, China; 2 School of Civil Engineering, Xi’an Railway Vocational & Technical Institute, Xi’an 710600, China; 3 MOE Key Laboratory of High-Speed Railway Engineering, School of Civil Engineering, Southwest Jiaotong University, Chengdu 610031, China

**Keywords:** expansive soil, cement, montmorillonite adsorption modifier, composite stabilization, swelling–shrinkage behavior, unconfined compressive strength

## Abstract

To enhance the strength and water stability of stabilized expansive soil, this study investigates the use of cement, montmorillonite adsorption modifier (MAM), and their composite system. Laboratory tests evaluated compaction characteristics, swell–shrink behavior, and mechanical performance. The results show that MAM more effectively regulates compaction by reducing optimum water content and increasing maximum dry density; 6% MAM increases maximum dry density by ≈0.04 g/cm^3^ and reduces optimum water content by ≈2%. In terms of swell–shrink behavior, MAM reduces both swelling and linear shrinkage more effectively than cement. The incorporation of 5% MAM reduces the free swelling ratio by 40% and the equilibrium moisture absorption by 2.7%, lowering the swelling classification to non-expansive. Furthermore, 5% MAM decreases the unloaded and loaded swelling ratio by 14.7% and 5%, respectively, while increasing MAM from 2% to 6% further reduces linear shrinkage by 1.12%. Cement significantly enhances compressive strength, with 7–28 d values reaching 2.2–2.7 times those of untreated soil at 9% content; however, its water stability under wet–dry cycles is limited. In contrast, the cement–MAM composite system achieves balanced improvement by simultaneously suppressing swelling and enhancing both strength and water stability. These findings provide a reference for the treatment and engineering application of expansive soils.

## 1. Introduction

Expansive soils, widely distributed and of significant geotechnical concern, are predominantly composed of strongly hydrophilic clay minerals such as montmorillonite and illite [1]. The charged surfaces and large specific surface areas of these minerals impart high water sensitivity [2,3]. Consequently, expansive soils undergo rapid swelling upon wetting, shrink upon drying, and develop cracks, exhibiting pronounced swelling–shrinkage cycles under periodic moisture variations. Long-term repeated volumetric changes can induce engineering hazards, including uneven subgrade settlement, slope instability, and structural cracking, particularly in arid and semi-arid regions [1,4,5]. Expansive soil is widely recognized as a typical problematic soil distributed across many countries, often causing engineering hazards and significant economic losses [6]. In China, expansive soils are extensively distributed, and numerous engineering problems have been reported since the construction of the Chengdu–Chongqing Railway [7]. In the United States, expansive soils cover approximately 25% of the land area, and since the failure of the steel siphon bridge project in 1938, they have continued to cause substantial engineering damage and economic loss [7,8]. To mitigate such risks, excavated expansive soils are often transported offsite and replaced with natural sand and gravel [9]. However, this approach increases transportation and disposal costs, generates dust, occupies land, and may induce ground settlement, vegetation degradation, and landslides [10,11]. Additionally, dependence on natural aggregates intensifies resource consumption and environmental pressure. Consequently, in situ stabilization of expansive soils through physical or chemical methods, aimed at reducing swelling potential while enhancing mechanical properties, has emerged as a critical strategy in geotechnical engineering, with important implications for structural safety and service life performance [12].

Cement, as a traditional stabilizer, has been widely used in expansive soil improvement engineering [13]. Its improvement mechanism mainly originates from hydration and pozzolanic reactions, producing calcium silicate hydrate, acicular hydrates, and a small amount of carbonates. These cementitious products can fill soil pores, wrap and bind particles, and gradually form a continuous rigid skeleton structure, transforming the soil from a dispersed state into a dense whole, thereby significantly improving bearing capacity [14]. Existing studies have shown that cement improvement has a significant effect on strength enhancement. For example, Wang et al. [15] pointed out that cement can significantly increase unconfined compressive strength; Yang et al. [16] found that when the cement content is 9%, the soil strength can be increased nearly tenfold; and Tang et al. [17] further showed that, compared with lime and fly ash, cement has a more significant effect on the strength improvement of expansive soil in Nanning. However, cement stabilization still has certain limitations [18]. Especially for expansive soils with high clay content and large specific surface area, a large amount of water is required to form bound water films, while cement has limited ability to regulate the thickness of these films [19]. As a result, its effectiveness in improving soil hydrophilicity and controlling swell–shrink behavior remains relatively insufficient [1,9]. Compared with some modern stabilizers that can directly modify the physicochemical properties of clay particles, cement mainly enhances strength but lacks targeted regulation of the soil–water interaction [20,21]. To effectively control swelling–shrinkage behavior, a relatively high cement content is usually required, which may bring additional economic and environmental burdens [22,23,24,25]. In addition, under environmental actions such as wet–dry cycles, the internal stress generated by swelling and shrinkage deformation can easily lead to structural deterioration of cement-treated soil, thereby reducing its water stability [26]. To compensate for these shortcomings, the use of modifier capable of regulating the surface charge of clay particles and reducing bound water films, namely the montmorillonite adsorption modifier (MAM), may be a feasible solution [27,28].

MAM is a weakly acidic resin (pH 6–7) belonging to organic chemical products, characterized by non-corrosive and non-polluting properties under normal use conditions. It can effectively improve the swell–shrink behavior of expansive soil while requiring minimal adjustment to construction procedures, with potential for application in green geotechnical engineering [29]. Its stabilization mechanism is primarily based on cation exchange, whereby high-valence cations (such as Al^3+^) replace low-valence cations (such as Na^+^, K^+^, and Ca^2+^) on the surface of soil particles. This process compresses the diffuse double layer and thins the adsorbed water film, thereby reducing soil hydrophilicity and mitigating swell–shrink behavior [27,30]. Laboratory investigations have confirmed MAM’s efficacy. Yu et al. [29] demonstrated that MAM significantly diminishes hydrophilicity and suppresses swelling–shrinkage in medium-expansive soils, while Yu et al. [27] reported that MAM-stabilized soils satisfy swelling–shrinkage criteria and exhibit excellent water stability under both wet and long-term conditions. By leveraging complementary mechanisms, combining cement with MAM for expansive soil stabilization can achieve synergistic optimization of structural reinforcement and interfacial control. Specifically, cement hydration products form a skeleton that enhances strength, while MAM reduces hydrophilicity to suppress swelling–shrinkage behavior. This combined approach can potentially realize high strength and low swelling with reduced cement usage and carbon emissions, thereby improving engineering performance, sustainability, and economic efficiency. However, most existing studies focus primarily on single modification methods, and research on the composite stabilization of MAM with traditional cementitious materials remains limited.

In this study, a representative expansive soil from Nanning, Guangxi, was selected to compare cement stabilization, MAM stabilization, and cement–MAM composite stabilization. Laboratory tests—including compaction, free swelling ratio, equilibrium moisture absorption, loaded and unloaded swelling ratios, shrinkage, and unconfined compressive strength (before and after wet–dry cycles)—were conducted to systematically evaluate the soils’ compaction characteristics, swelling potential grade, swelling–shrinkage behavior, mechanical performance, and water stability. The results provide a basis for assessing the feasibility of cement–MAM composite stabilization and guiding the design and implementation of green subgrade fills in expansive soil regions.

## 2. Materials and Methods

### 2.1. Materials

The raw materials used in the expansive soil modification tests included expansive soil, cement, water, and MAM. The basic physical properties of the soil are summarized in Table 1. The mass fractions of particle sizes within (0, 2), [2, 5), [5, 75), and [75, 100] μm are 41.6%, 27.0%, 30.0%, and 1.4%, respectively. Based on a free swelling ratio of 60–90% and an equilibrium moisture absorption of 4.8–6.8%, and in accordance with JTG C20-2011, the soil is classified as medium expansive soil [31]. In addition, more than 50% of the particles are smaller than 75 μm, the liquid limit exceeds 50%, and the plasticity index is 46.9, which is greater than 0.73 (74.9 − 20) = 40. According to the Unified Soil Classification System (USCS), the soil is classified as fat clay (CH) [32]. Furthermore, the activity index is 1.13, falling within the range of 0.75–1.25, indicating normal clay [33].

The curing agent used in the tests was PC42.5 composite Portland cement, whose main components were SiO_2_, Fe_2_O_3_, Al_2_O_3_, and CaO. The initial and final setting times were 195 min and 250 min, respectively. In the cement particle size distribution, the mass fractions of particles in the ranges of 0–3 μm, 3–45 μm, and 45–80 μm were 36.20%, 36.70%, and 26%, respectively. Tap water was used in the tests.

The MAM used in the tests was a water-soluble ion-exchange resin, and its modification mechanism was primarily based on ion exchange. MAM can dissociate in water to produce high-valence cations (X^n+^) and anions (Y^n−^), which participate in the ion exchange process [27]. Taking aluminum ions as an example, the reaction process can be described as follows, and the overall reaction equation is shown in Equation (1).

(1) $${\left[\mathrm{soil}\;\mathrm{particles}\right]}^{n-}{\left[M\right]}^{+}+{\left[X\right]}^{n+}+{\left[Al\right]}^{3+}+{\left[Y\right]}^{n+}\to {\left[\mathrm{soil}\;\mathrm{particles}\right]}^{n-}{\left[Al\right]}^{3+}+{\left[Y\right]}^{n-}+{\left[X\right]}^{n+}+{\left[M\right]}^{+}$$

After low-valence cations are replaced by high-valence cations, the thickness of the diffuse double layer and the bound water film on the surface of expansive soil particles decreases. This reduces the water required to form surface water films, thereby weakening soil hydrophilicity and reducing swell–shrink behavior.

### 2.2. Experimental Program

#### 2.2.1. Test Mix Proportion

In this study, compaction, free swelling ratio, equilibrium moisture absorption, loaded and unloaded swelling ratio, shrinkage, and unconfined compressive strength tests before and after dry–wet cycles were conducted to systematically analyze the compaction characteristics, expansibility grade, expansibility, shrinkage behavior, strength, and water stability of different stabilized soils, thereby evaluating the feasibility of cement–MAM composite modification. Here, the cement content was expressed as the mass ratio of cement to dry soil, denoted as C-S, whereas the MAM content was expressed as the mass ratio of MAM to dry soil, denoted as MAM-S.

To compare different modification methods, preliminary tests of cement-only and MAM-only stabilization were conducted, focusing on equilibrium moisture absorption, free swelling ratio, and compressive strength. For cement-only tests, C-S ranged from 1% to 7% with a 2% increment. At C-S = 7%, the free swelling ratio and equilibrium moisture absorption were 43.2% and 3.4%, respectively, slightly above the non-expansive range, while the 28-d compressive strength reached 1542 kPa. For MAM-only tests, MAM-S ranged from 2% to 7% with a 1% increment. At MAM-S = 5%, the free swelling ratio and equilibrium moisture absorption were 38% and 2.5%, respectively, corresponding to a critical state between expansive and non-expansive soil. At MAM-S = 6%, these values decreased to 34% and 2.39%, indicating non-expansive behavior. Considering that MAM mainly regulates swell–shrink characteristics, MAM-S = 6% was selected for subsequent tests. Although the compressive strength at C-S = 7% meets most backfill requirements [9], its swelling potential remains higher than that of non-expansive soil; therefore, C-S was increased to 9% in the formal tests. The tests were divided into four groups: the control group with untreated soil (C-S = 0, MAM-S = 0); the cement-stabilized group with C-S ranging from 1% to 9% (MAM-S = 0); the MAM-stabilized group with MAM-S ranging from 2% to 6% (C-S = 0); and the composite-stabilized group with C-S ranging from 1% to 9% and MAM-S ranging from 2% to 6%. The detailed experimental program is presented in Table 2.

#### 2.2.2. Testing Procedure and Methods

**Figure 1:**
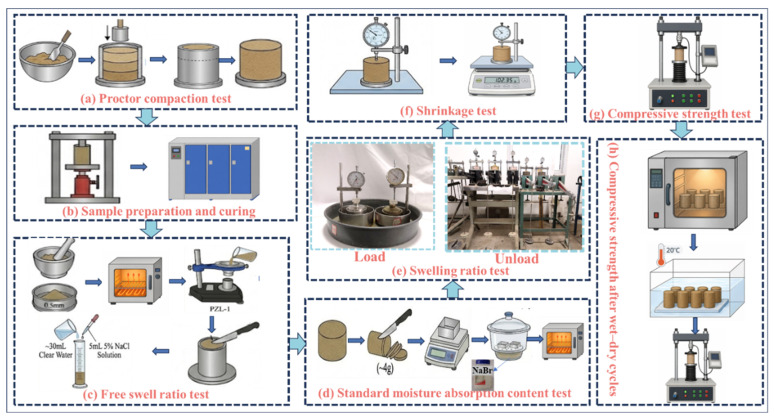
Testing procedure.

In this study, the testing procedure is shown in Figure 1.

The compaction test is essential for determining the compaction characteristics of stabilized soil, which directly influence compaction quality and engineering performance [34]. First, the optimum water content (*w*_opt_) and maximum dry density (*ρ*_dmax_) are determined. Compacted specimens are then prepared at a compaction degree of 0.92 and cured under standard conditions (20 ± 2 °C, relative humidity ≈ 95%) for subsequent tests. The swelling grade is initially evaluated to assess the improvement effect. Subsequently, loaded and unloaded swelling tests, along with shrinkage tests, are conducted to analyze swell–shrink behavior and mitigate settlement and cracking caused by volume changes. Finally, unconfined compressive strength tests before and after wet–dry cycles are performed to evaluate bearing capacity and water stability. The specific test methods are as follows:

(1) Compaction test: Water, cement, and MAM were added to the soil and thoroughly mixed. In accordance with JTG D3430-2020 [35], a DM-II compaction apparatus (Beijing Zhongke Lanjian Instrument Equipment Co., Ltd., Beijing, China) was used to perform a light compaction test. The hammer mass was 4.5 kg with a drop height of 45 cm. The soil was compacted in five layers, with 27 blows per layer, corresponding to a total compaction energy of approximately 2.68 kJ. The test was completed within 1 h, as shown in Figure 1a. Five specimens with varying water contents were prepared, with increments of 1–3%. After uniform mixing, the samples were sealed and left overnight before testing. The dry density–water content curve was obtained by plotting dry density against water content, with the peak indicating the maximum dry density and optimum water content. If no clear peak is observed, additional data points should be added or the test repeated.

(2) Free swelling ratio test: According to JTG D3430-2020, the sample for the free swelling ratio (*F_S_*) test was first crushed and passed through a 0.5 mm sieve, then dried at 105–110 °C for at least 8 h and cooled to room temperature in a desiccator. As shown in Figure 1c, a funnel was placed above the measuring cup of the PZL-1 tester (Beijing Zhongke Luda Testing Instrument Co., Ltd., Beijing, China), and the soil was filled to the initial volume *V*_0_. The sample was then transferred into a measuring cylinder containing sodium chloride solution, and the soil volume was recorded every 2 h until stabilization at V_1_. The free swelling ratio was calculated according to Equation (2). The test was conducted in parallel twice, and the average value was taken. When *F_S_* ≥ 60%, the allowable difference between parallel results should not exceed 8%; when *F_S_* < 60%, it should not exceed 5%; otherwise, the test was repeated.

(2) $$F_{s}=\frac{V_{1}-V_{0}}{V_{0}}$$

(3) Equilibrium moisture absorption test: The equilibrium moisture absorption reflects the ability of soil to absorb moisture under a constant humidity environment and is an important indicator for evaluating the hydrophilicity, hygroscopic sensitivity, and swell–shrink potential of expansive soil. This parameter characterizes the response of soil to environmental humidity variations. A higher equilibrium moisture absorption indicates stronger surface adsorption capacity of soil particles, a greater amount of weakly bound water, and a thicker diffuse double layer, thereby resulting in a higher potential for moisture-induced swelling and shrinkage [9]. According to JTG D3430-2020, approximately 4 g of flake-shaped soil samples cured for 7 d were taken and placed in an aluminum box with a mass of m_1_. The box was then placed in a sealed desiccator containing saturated sodium bromide solution. The mass was measured daily until stabilization and recorded as m_2_, as shown in Figure 1d. The sample was subsequently oven-dried to constant weight to obtain m_3_. The equilibrium moisture absorption (*w_a_*) was then calculated according to Equation (3). The test was conducted in parallel twice, and the allowable error between results should not exceed 0.2%; otherwise, the test was repeated, and the average value was taken.

(3) $$w_{a}=\frac{m_{2}-m_{3}}{m_{3}-m_{1}}$$

(4) Unloaded swelling ratio test: According to JTG D3430-2020, after 7 d of curing, the sample was trimmed into a cylindrical specimen with a height of 2 cm using a ring cutter, and the unloaded swelling ratio (*δ_e_*) was measured using a WZ-2 tester (Nanjing Soil Instrument Factory Co., Ltd., Nanjing, China) (see Figure 1e). The instrument has a measurement accuracy of 0.01 mm and was zero-calibrated before testing to ensure a stable initial dial gauge reading. During the test, a wetted porous stone was placed on the base so that the bottom surface of the specimen was in full contact with it and immersed in a water bath. A dial indicator was positioned at the center of the top surface of the specimen and set to zero to monitor deformation. Throughout the test, the water level was maintained flush with the bottom surface of the specimen, and deformation data were recorded at predetermined time intervals (from 10 min to 24 h) until no further deformation occurred. *δ_e_* was calculated according to Equation (4). The test was conducted in parallel twice, and the average value was taken. The allowable difference between parallel results should not exceed 1% when *δ_e_* ≥ 10% and 0.5% when *δ_e_* < 10%. Otherwise, the test was repeated.

(4) $$\delta_{e}=\frac{R_{t}-R_{0}}{H_{0}}\times 100$$

where *R_t_* is the dial indicator reading at time *t*, *R*_0_ is the dial indicator reading at the beginning of the test, and *H*_0_ is the initial height of the specimen.

(5) Loaded swelling ratio test: According to JTG D3430-2020, an overburden pressure of 25 kPa was applied in the loaded swelling ratio (*δ_ep_*) test, which was conducted using a WG-3C consolidation apparatus (Nanjing Ningxi Soil Instrument Co., Ltd., Nanjing, China). Displacement was measured using a dial gauge with an accuracy of 0.01 mm. Zero calibration was performed before testing to ensure stable and reliable initial readings. Meanwhile, the pressure measurement system was calibrated in accordance with laboratory metrology standards. The specimen preparation method was the same as that used for the *δ_e_* test. The specimen was placed into the container, followed by the sequential placement of the porous stone and cover plate. A dial indicator was then installed, and an initial load of 1 kPa was applied, after which the initial reading was recorded. The load was subsequently applied in stages. After deformation became stable, water was added into the container until the water level was approximately 5 mm above the top surface of the specimen, allowing the specimen to be soaked from bottom to top. During soaking, the dial indicator reading was recorded every 2 h until the difference between two consecutive readings did not exceed 0.01 mm. After the test, the load was removed and the specimen was taken out. *δ_ep_* was calculated according to Equation (5). The test was conducted in parallel twice, and the average value was taken. The allowable difference between parallel results should not exceed 1% when *δ_ep_* ≥ 10% and 0.5% when *δ_ep_* < 10%; otherwise, the test was repeated.

(5) $$\delta_{ep}=\frac{R_{t}+R_{P}-R_{0}}{H_{0}}\times 100$$

where *H*_0_ is the initial height of the specimen (mm), *R_t_* is the dial indicator reading (mm) after the specimen swelling has stabilized under the load P, *R_P_* is the compressive deformation of the soil specimen under the load P, and *R*_0_ is the dial indicator reading before loading.

(6) Shrinkage test: According to JTG D3430-2020, after 7 d of curing, the specimen was saturated using the immersion method, and the vertical linear shrinkage (*e_sL_*) was measured using a WZ-2 shrinkage apparatus (Wuxi Serve Real Technology Co., Ltd., Wuxi, China) equipped with a dial gauge, as shown in Figure 1f. The test was conducted at room temperature (≤30 °C). At the initial stage, the dial indicator reading and specimen mass were recorded every 8 h; after 2 d, the measurements were taken once every 12 h until the dial indicator reading remained unchanged for three consecutive measurements. The *e_sL_* and water content (*w_sL_*) were calculated according to Equation (6) and Equation (7), respectively.

(6) $$e_{sL}=\frac{R_{t}-R_{0}}{H_{0}}\times 100$$

where *e_sL_* is the linear shrinkage ratio, *H*_0_ is the original height of the specimen, *R_t_* is the dial gauge reading at a given moment during shrinkage, and *R*_0_ is the initial dial indicator reading.

(7) $$w_{sL}=(\frac{m_{t}}{m_{s}}-1)\times 100$$

where *w_sL_* is the water content at a given time, *m_t_* is the specimen mass measured at that time, and *m_s_* is the dry soil mass.

When the dial indicator reading remains unchanged for three consecutive measurements, the specimen is placed in an oven at 105–110 °C and dried to a constant mass, after which the water content is determined. A relationship curve is then plotted with *e_sL_* as the vertical coordinate and *w_sL_* as the horizontal coordinate. The tangents to the linear shrinkage section and the horizontal section corresponding to shrinkage stabilization are drawn, respectively, and the water content corresponding to the intersection of these two straight lines is taken as the shrinkage limit (*w_s_*). The test was conducted in parallel twice. When the ratio of the difference between the larger and smaller values to the smaller value satisfies the following criteria, the average value is taken as *e_sL_*: it should not exceed 5% when *e_sL_* ≥ 10% and 3% when *e_sL_* < 10%. Otherwise, the test was repeated.

(7) Unconfined compressive strength test: Effective control of expansion characteristics is a prerequisite for the engineering application of expansive soils, and the compressive strength must also meet the design requirements to ensure adequate bearing capacity [36]. According to JTG D3430-2020, after standard curing (20 ± 2 °C and a relative humidity of 95%) for 7 d and 28 d, the specimens were subjected to unconfined compressive strength testing (*q_u_*) using a TLD-127SD testing machine (Shandong Luda Testing Instrument Co., Ltd., Cangzhou, China). The maximum load capacity of this machine is 200 kN, and two loading rates, 50 mm/min and 1 mm/min, are available. A loading rate of 1 mm/min was adopted in this study, as shown in Figure 1g. Six specimens were prepared for each mix proportion. When both the standard deviation and its ratio to the mean are within 10%, the average value is taken as the result; otherwise, the test was repeated.

Considering the disintegration observed in the cement-stabilized group during the shrinkage test, as well as the dry–wet cycles that may be encountered in practical engineering, the test was conducted with reference to ASTM D559 [37]. After curing for 7 d, the specimens were dried in an oven at 70 °C for 11 h, then removed and left to stand for 1 h, and subsequently immersed in clean water at 20 °C for 12 h, which constituted one dry–wet cycle, as shown in Figure 1h. A total of 9 cycles were performed, after which the unconfined compressive strength (*q_uw_*) was measured. Similarly, six specimens were prepared for each mix proportion. When the standard deviation relative to the mean does not exceed 10%, the average value is taken as the test result; otherwise, the test was repeated.

## 3. Results and Analysis

### 3.1. Compaction Characteristics

**Figure 2:**
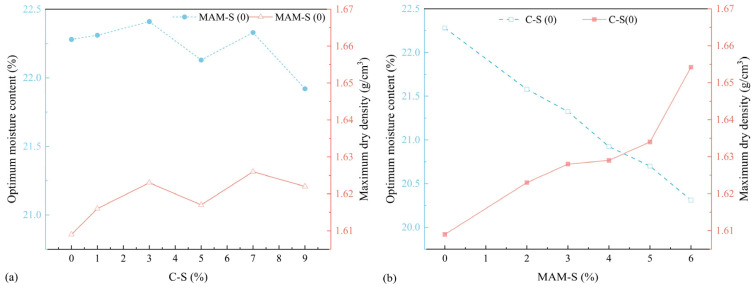
Optimum moisture content and maximum dry density of the single-stabilized groups: (**a**) variation with C-S; (**b**) variation with MAM-S.

The compaction test results are shown in Figure 2. As illustrated in Figure 2a, when the cement content (C-S) increases from 0% to 9%, the optimum water content (*w*_opt_) slightly decreases from 22.3% to 21.9%, while the maximum dry density (*ρ*_dmax_) increases from 1.61 g/cm^3^ to 1.62 g/cm^3^. The variation is limited, indicating that cement has a relatively minor effect on compaction characteristics [38]. In contrast, the MAM-treated group shows a more pronounced trend. As MAM-S increases from 0% to 6%, (*w*_opt_) decreases approximately linearly from about 22.3% to 20.3%, while (*ρ*_dmax_) increases from 1.61 g/cm^3^ to above 1.65 g/cm^3^. This behavior is attributed to the fine particle size and large specific surface area of expansive soil, which promote water adsorption and the formation of bound water films, resulting in strong hydrophilicity. After adding MAM, Al^3+^ replaces low-valence cations such as Na^+^ and K^+^ on particle surfaces, compressing the diffuse double layer and thinning the bound water film. This reduces water adsorption and the water required to form surface films, thereby weakening soil hydrophilicity and decreasing (*w*_opt_) [39]. Meanwhile, the thinner bound water film increases the solid phase proportion per unit volume, leading to higher *ρ*_dmax_. Although Ca^2+^ from cement hydration can also participate in cation exchange, its effect is weaker than that of MAM [7,27,29,36,40].

**Figure 3:**
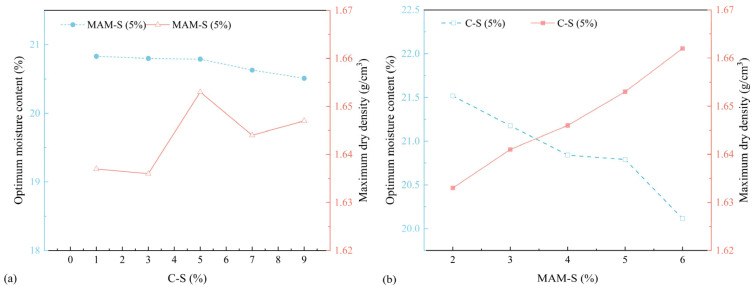
Optimum moisture content and maximum dry density of the composite-stabilized groups: (**a**) variation with C-S; (**b**) variation with MAM-S.

In the composite stabilization group (see Figure 3), with MAM-S at 5%, *w*_opt_ decreases with increasing C-S, and *ρ*_dmax_ exhibits variable trends. When C-S is 5%, the trends and magnitudes of *w*_opt_ and *ρ*_dmax_ with changing MAM-S are similar to those at C-S of 0%, indicating minimal interaction between C-S and MAM-S regarding compaction behavior.

### 3.2. Swelling Potential

**Figure 4:**
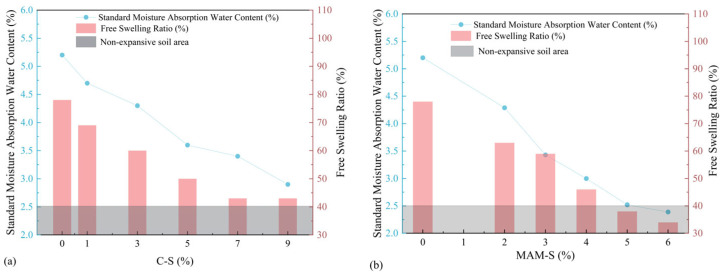
Free swelling ratio and equilibrium moisture absorption of the single-stabilized groups: (**a**) variation with C-S; (**b**) variation with MAM-S.

When the free swelling ratio (*F_S_*) is lower than 40% and the equilibrium moisture absorption (*w_a_*) is lower than 2.5%, the soil can be regarded as having no significant swelling potential and is therefore classified as non-expansive soil. Figure 4 shows that, in the cement-stabilized group, both *F_S_* and *w_a_* decreased with increasing cement content (C-S). This is because Ca^2+^ released during cement hydration exchanges with low-valence cations such as Na^+^ and K^+^ on soil particle surfaces, compressing the diffuse double layer and thinning the bound water film. This process reduces water adsorption and the amount of water required for bound water film formation, thereby weakening soil hydrophilicity and inhibiting swelling deformation [9,29,39,40]. When C-S reached 9%, *F_S_* decreased from 78% to 43% and *w_a_* decreased from 5.20% to 2.9%, indicating that the soil could be classified as weak expansive soil. In the MAM-stabilized group, *F_S_* and *w_a_* decreased significantly with increasing MAM content (MAM-S). When MAM-S reached 6%, *F_S_* decreased to 34% and *w_a_* decreased to 2.39%, and the soil could thus be classified as non-expansive soil, indicating that MAM exerted a more pronounced inhibitory effect on swelling potential [4,27,29,41].

**Figure 5:**
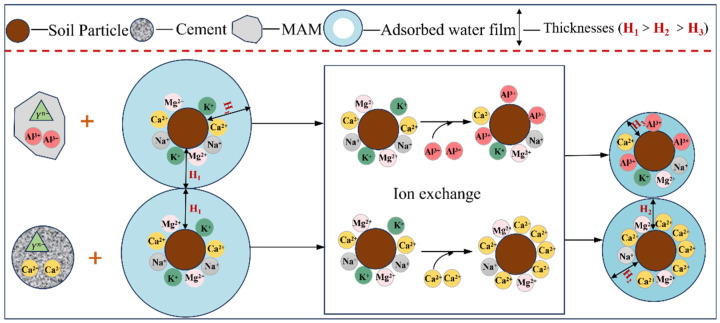
MAM and cement reduce the thickness of the bound water film.

**Figure 6:**
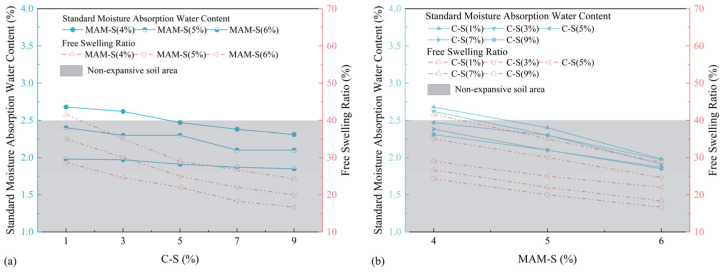
Free swelling ratio and equilibrium moisture absorption of the composite-stabilized group: (**a**) variation with C-S; (**b**) variation with MAM-S.

This is consistent with the mechanism of decreasing optimum water content and increasing maximum dry density. This is because the Al^3+^ released by MAM can not only replace Na^+^ and K^+^, but also exchange with Ca^2+^ and Mg^2+^, thereby enhancing the Coulomb interaction between particles and further weakening hydrophilicity [9,29,39,40,42]. After the exchange between high- and low-valence ions, the interparticle Coulomb force is enhanced, leading to a thinner bound water film on particle surfaces and reduced soil hydrophilicity, consistent with findings in the literature [9,29,39,40,42]. As a result, its improvement effect is superior to that of cement, as shown in Figure 5 [43]. As can be seen from Figure 6, in the composite-stabilized group, the variation trends of *F_S_* and *w_a_* were generally consistent with those in the single-stabilized groups. When MAM-S was 5% and C-S was 1%, *F_S_* was about 35% and *w_a_* was about 2.4%, and the soil could already be classified as non-expansive soil. After physical or chemical modification of expansive soil, the required cement content to achieve a non-expansive state can be reduced. Du et al. [9] reported that sand replacement effectively lowers the cement demand for meeting non-expansive soil criteria. In this study, compared with C-S = 9%, the composite modification reduced the cement content required to achieve the same swelling grade by more than 8%.

### 3.3. Swelling Ratio

**Figure 7:**
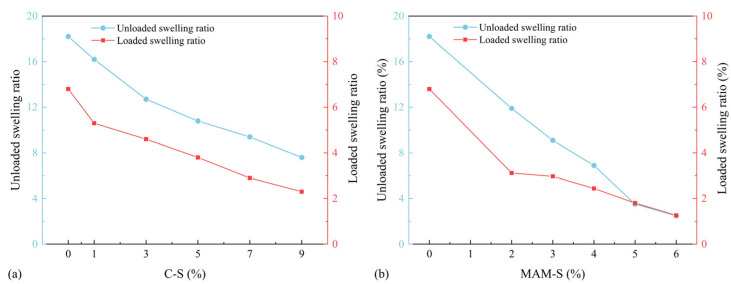
Unloaded swelling ratio and loaded swelling ratio of the single-stabilized groups: (**a**) variation with C-S; (**b**) variation with MAM-S.

The swelling ratio reflects the degree of volumetric expansion deformation of compacted stabilized soil under water immersion conditions and is used to evaluate its volumetric stability upon wetting [44]. The test results for the single-stabilized groups are shown in Figure 7. From a mechanistic perspective, upon water absorption, expansive soils form bound water films on particle surfaces, increasing interparticle spacing and causing volumetric expansion. In cement-stabilized systems, swelling is jointly suppressed by ion exchange and hydration reactions. Hydration products gradually develop and constrain soil deformation, thereby inhibiting expansion, and this process is time-dependent due to the long-term nature of hydration. In contrast, MAM introduces higher-valence cations, which more effectively replace low-valence ions on particle surfaces, leading to stronger compression of the diffuse double layer and more significant reduction in bound water film thickness. As a result, the hydrophilicity of clay particles is further reduced, and swelling is more effectively suppressed. As shown in Figure 7, MAM exhibits a greater reduction in swelling ratio [29,39,42]. In the cement-stabilized group, when the cement content (C-S) was 5%, the unloaded swelling ratio (*δ_e_*) decreased from 18.2% to 10.8%, a reduction of 7.4 percentage points, while the loaded swelling ratio (*δ_ep_*) decreased from 6.8% to 3.8%, a reduction of 3.0 percentage points. In the MAM-stabilized group, when the MAM content (MAM-S) was 5%, *δ_e_* decreased from 18.2% to 3.5%, a reduction of 14.7 percentage points, whereas *δ_ep_* decreased from 6.8% to 1.8%, a reduction of 5.0 percentage points. This indicates that, at the same dosage, the inhibitory effect of MAM on the swelling ratio was markedly superior to that of cement.

**Figure 8:**
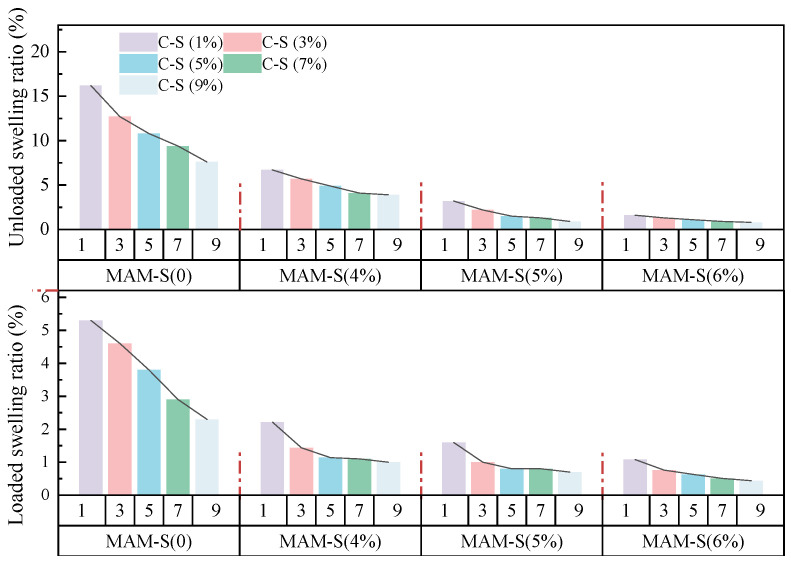
Unloaded swelling ratio and loaded swelling ratio of the composite-stabilized group.

In the composite-stabilized group (see Figure 8), when MAM-S was fixed, increasing C-S further reduced the swelling ratio. However, when MAM-S was relatively high, the decrease in swelling ratio became less pronounced with increasing C-S. This is because, at higher MAM-S levels, the swelling ratio itself had already been reduced to a relatively low level, while the ionic interactions between cement and MAM around the soil particles may also have mutually influenced each other [7]. In addition, in the composite-stabilized groups with different MAM-S values, the swelling ratio decreased significantly with increasing MAM-S. When the MAM content was 6% and the cement content was 1%, *δ_e_* was 1.6%, which was far lower than the value of 7.6% corresponding to a cement content of 9% in the cement-stabilized group. These results indicate that the addition of MAM on the basis of cement modification can significantly enhance the improvement effect on the swelling ratio of expansive soil, thereby improving the volumetric stability of compacted fills in engineering applications.

### 3.4. Shrinkage Behavior

**Figure 9:**
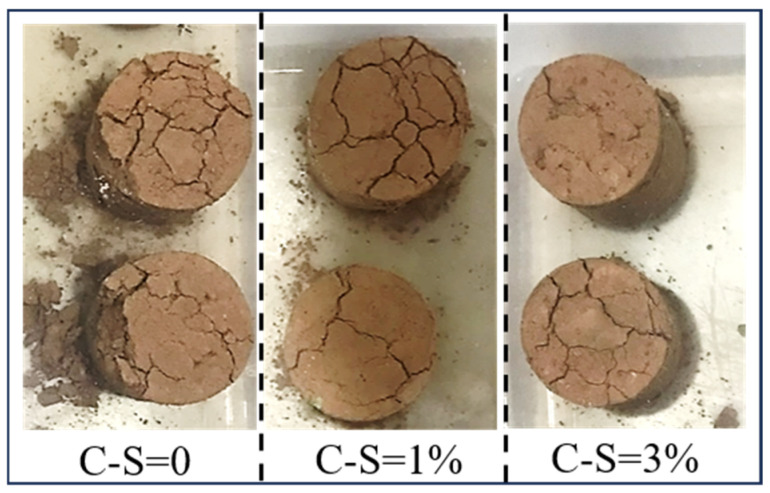
Disintegration of specimens upon water saturation.

**Figure 10:**
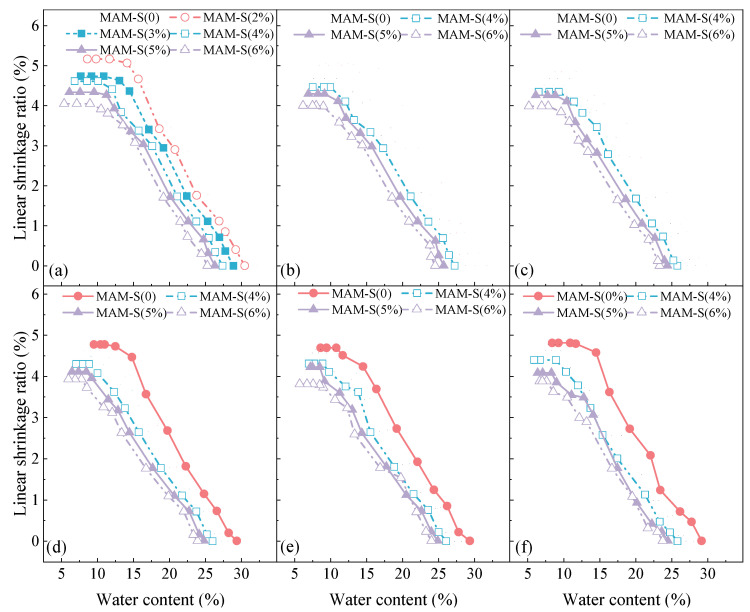
Relationship between linear shrinkage ratio and water content of stabilized expansive soil: (**a**) C-S = 0; (**b**) C-S = 1%; (**c**) C-S = 3%; (**d**) C-S = 5%; (**e**) C-S = 7%; (**f**) C-S = 9%.

The shrinkage test is used to determine indices such as the linear shrinkage ratio and shrinkage limit of expansive soil under conditions without lateral restraint or overlying load [45]. The relationship curves between the linear shrinkage ratio and water content of the stabilized expansive soils are shown in Figure 9. When the MAM content was 0, the specimens with C-S of 0, 1%, and 3%, respectively, all disintegrated during water saturation, and the corresponding data could therefore not be obtained. This is because, at low cement content, the specimen retains strong hydrophilicity. Upon water immersion, it absorbs water and expands, generating internal swelling stress that promotes crack initiation and propagation. Once formed, cracks facilitate water infiltration, and with continued immersion, they progressively penetrate the specimen, accelerating particle separation and local collapse, ultimately leading to gradual disintegration. This also indicates that the use of cement alone for expansive soil modification may be associated with insufficient water stability. As can be seen from Figure 10, for the specimens that did not disintegrate during water immersion, the variation trends of the linear shrinkage ratio with water content were basically consistent among all groups. As the water content decreased, the linear shrinkage ratio first increased linearly, then its rate of increase gradually slowed, and finally tended to stabilize. During water loss from the expansive soil, the loss of water between particles and capillary action reduced the interparticle spacing, causing the linear shrinkage ratio to increase with increasing water loss. Once a stable particle skeleton had formed and the capillary water had basically disappeared, the remaining water no longer affected the interparticle spacing. Therefore, even if further water loss occurred, the soil volume tended to remain stable [29,46,47].

The shrinkage indices of each group are presented in Table 3. In the cement-stabilized group, the specimens did not disintegrate when the cement content reached 5%. However, as the cement content increased from 5% to 9%, no consistent trend was observed in either the linear shrinkage ratio or the shrinkage limit. This may be attributed to the following reason: although cement can reduce the hydrophilicity of expansive soil, which is beneficial for lowering the linear shrinkage ratio, its hydration is a long-term process. During water saturation, cement hydration may induce chemical shrinkage, thereby aggravating the overall shrinkage. Zhang et al. [48] provided a relatively detailed discussion on the shrinkage associated with cement hydration reactions in their study. Therefore, the variation in linear shrinkage ratio depends on the relative dominance between the ionic interactions induced by cement and the hydration reaction during water saturation [48,49]. In the MAM-stabilized group, the specimens did not disintegrate when 2% MAM was added, and as the MAM content increased from 2% to 6%, the linear shrinkage ratio and shrinkage limit decreased by 1.12% and 2.71%, respectively. This indicates that, under the same 4% increase in dosage, MAM was far more effective than cement in improving the shrinkage behavior of expansive soil. In the composite-stabilized group, when the cement content was 9% and the MAM content increased from 2% to 6%, the linear shrinkage ratio and shrinkage limit decreased by 0.66% and 1.74%, respectively, with the reductions being about 0.6 times those in the single-stabilized groups. This is mainly because the linear shrinkage ratio and shrinkage limit in the composite-stabilized group were already at relatively low levels, while the ionic interactions between cement and MAM among soil particles may have exerted mutual inhibition, thereby weakening their respective improvement effects on the shrinkage behavior of expansive soil [29,36,41,46,47].

### 3.5. Unconfined Compressive Strength

#### 3.5.1. Without Dry–Wet Cycles

**Figure 11:**
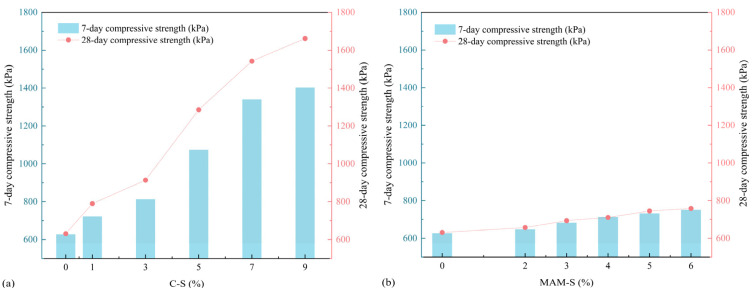
Unconfined compressive strength of the single-stabilized groups: (**a**) variation with C-S; (**b**) variation with MAM-S.

The 7-day (*q*_u7_) and 28-day (*q*_u28_) unconfined compressive strengths of the specimens are presented in Figure 11. As shown in Figure 11a, in the cement-stabilized group, *q*_u7_ and *q*_u28_ increase approximately linearly with cement content (C-S). When C-S reaches 9%, *q*_u7_ and *q*_u28_ are 1403 kPa and 1662 kPa, respectively, corresponding to 2.2 and 2.7 times the strengths of untreated soil. The strength enhancement in cement-stabilized soils is attributed to hydration products, including Ca(OH)_2_, calcium silicate hydrate (C-S-H) gel, calcium aluminate hydrate (C_3_AH_6_), and ettringite (AFt) [50]. These products interweave to form a network structure, filling interparticle voids and cementing soil particles to create a continuous load-bearing skeleton [14]. In the MAM-stabilized group, *q*_u7_ and *q*_u28_ also increase approximately linearly with MAM content (MAM-S), but the increment is substantially smaller than that of cement, as shown in Figure 11b.

**Figure 12:**
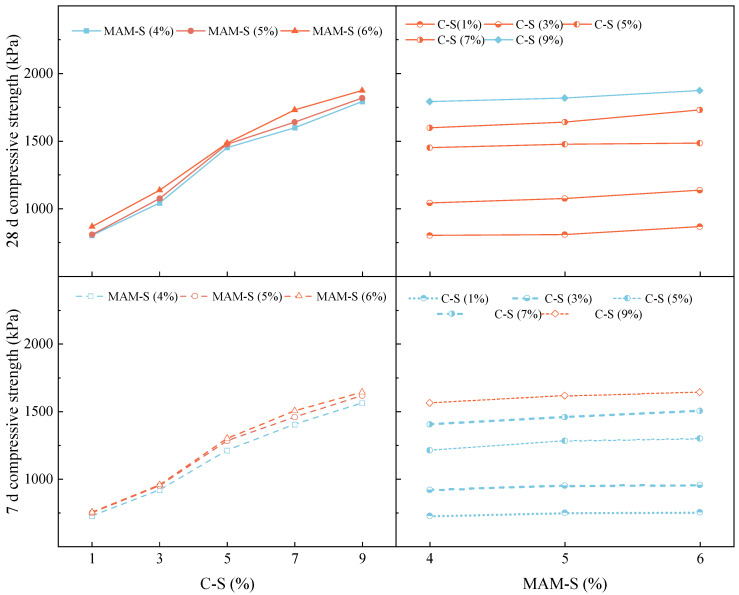
Unconfined compressive strength of the composite-stabilized group.

In the composite stabilization group, across varying MAM-S contents, the *q*_u7_ and *q*_u28_ of stabilized expansive soils show no significant change with increasing C-S, indicating that MAM addition does not affect the cement-induced strength gain [51]. Similarly, under different C-S levels, *q*_u7_ and *q*_u28_ exhibit no marked trend with increasing MAM-S, suggesting that cement addition does not influence the effect of MAM on the unconfined compressive strength of the stabilized soils, as shown in Figure 12.

Based on the approximately linear relationship of *q*_u7_ and *q_u_*_28_ with C-S and MAM-S, a predictive model for *q*_u7_ and *q*_u28_ was developed using the normalization method [36]. Since C-S is 0 for MAM-S values of 2% and 3%, these data points were excluded from the analysis. Therefore, combinations of C-S and MAM-S corresponding to MAM-S values of 0%, 4%, 5%, and 6% and C-S values of 0%, 1%, 3%, 5%, 7%, and 9% were selected for model development. Taking *q*_u7_ as an example, C-S is denoted as *X*_1_ and MAM-S as *X*_2_. Normalization factors *Xq*_u7_ (*X*_1_) and *Xq*_u7_ (*X*_2_) were introduced to quantify the respective effects of C-S and MAM-S on *q*_u7_, as expressed in Equations (8) and (9).

(8) $$X_{q_{u7}}\left(X_{1}\right)=\frac{q_{u7}\left(X_{1},X_{2}\right)-q_{u7}\left(0,X_{2}\right)}{q_{u7}\left(0.09,X_{2}\right)-q_{u7}\left(0,X_{2}\right)}$$

In the equations, *q*_u7_ (0.09, *X*_2_) and *q*_u7_ (0, *X*_2_) are the normalized characteristic strengths at X_1_ values of 0.09 and 0, respectively.

(9) $$X_{q_{u7}}\left(X_{2}\right)=\frac{q_{u7}\left(X_{1},X_{2}\right)-q_{u7}\left(X_{1},0\right)}{q_{u7}\left(X_{1},0.06\right)-q_{u7}\left(X_{1},0\right)}$$

In the equations, *q*_u7_ (*X*_1_, 0.06) and *q*_u7_ (*X*_1_, 0) are the normalized characteristic strengths at *X*_2_ values of 0.06 and 0, respectively.

By substituting the strength data *q*_u7_ (0,0), *q*_u7_ (0,0.06), *q*_u7_ (0.09,0), and *q*_u7_ (0.09,0.06) into Equations (8) and (9), the expression of *q*_u7_ in terms of the normalization factors is obtained, as shown in Equation (10).

(10) $$q_{u7}(X_{1},X_{2})=627+776X_{q_{u7}}\left(X_{1}\right)+123.8X_{q_{u7}}\left(X_{2}\right)+114.2X_{q_{u7}}\left(X_{1}\right)X_{q_{u7}}\left(X_{2}\right)$$

Using Equations (8) and (9) along with the strength data from all mixture proportions, the relationships of *Xq*_u7_ (*X*_1_) and *Xq*_u7_ (*X*_2_) as functions of *X*_1_ and *X*_2_ were fitted, as expressed in Equations (11) and (12).

(11) $$X_{q_{u7}}\left(X_{1}\right)=12.06X_{1}-0.05\;(R^{2}=0.978)$$

(12) $$X_{q_{u7}}\left(X_{2}\right)=16.75X_{2}-0.03\;(R^{2}=0.889)$$

By substituting Equations (11) and (12) into Equation (10), the expression for *q*_u7_ is obtained as Equation (13).

(13) $$q_{u7}=586.3+9325.8X_{1}+1981.2X_{2}+23071.3X_{1}X_{2}$$

Similarly, the expression for the 28-day unconfined compressive strength (*q*_u28_) is derived as Equation (14).

(14) $$q_{u28}=625.1+12048.6X_{1}+1977.3X_{2}+15388.5X_{1}X_{2}$$

To verify the accuracy of the models in Equations (13) and (14), the mix proportion parameters were substituted into the equations, and the coefficient of determination (*R*^2^), root mean square error (RMSE), and mean absolute error (MAE) were calculated. For Equation (13), *R*^2^, MAE, and RMSE are 0.978, 45.14, and 49.06, respectively, while for Equation (14), they are 0.982, 55.11, and 44.7. The *R*^2^ of Equation (13) exceeds 0.95, indicating good predictive accuracy. The MAE of approximately 45 kPa and the small difference between MAE and RMSE (within 5 kPa) suggest stable predictions without significant outliers. Overall, Equation (13) shows good predictive performance. Equation (14) also exhibits high accuracy, although larger deviations are observed compared with Equation (13).

#### 3.5.2. Dry–Wet Cycles

**Figure 13:**
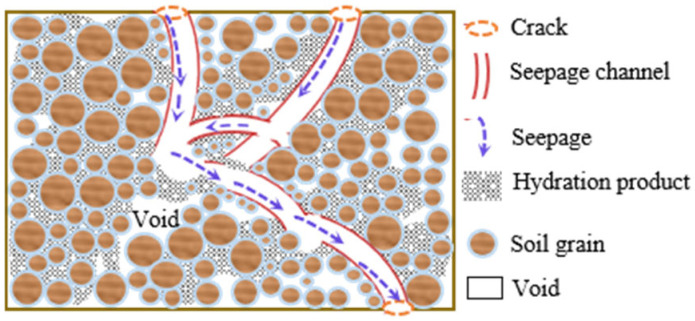
Moisture infiltration of specimens during wet–dry cycles.

The test results of the unconfined compressive strength of the specimens are presented in Table 4. As can be seen from Table 4, in the cement-stabilized group, all specimens disintegrated during the dry–wet cycles. During repeated drying and wetting, internal stresses induced by shrinkage and swelling promote crack initiation. With continued cycles, cracks propagate and connect with internal pores, forming seepage channels that facilitate water infiltration. This process weakens interparticle bonding, damages the microstructure, accelerates strength degradation, and reduces durability, as shown in Figure 13. In contrast, MAM reduces soil hydrophilicity through ion exchange, compresses the diffuse double layer, and thins the bound water film, thereby lowering swelling induced by water absorption. This effect mitigates swell–shrink deformation during wet–dry cycles and suppresses crack initiation and propagation [52,53,54]. In the MAM-stabilized group, the specimens disintegrated when the MAM content (MAM-S) was lower than 4%, whereas no disintegration occurred when MAM-S reached 5%. This is because, when MAM-S was 5%, the specimens had approached the state of non-expansive soil. In the composite-stabilized group, most specimens did not disintegrate. Notably, when the cement content (C-S) was 3%, a MAM-S of 4% markedly enhanced the water stability of the specimens and prevented disintegration. These results indicate that, on the basis of the strength enhancement provided by cement, the addition of MAM enabled the stabilized soil to maintain better bearing capacity under dry–wet cycles, thereby compensating for the insufficient water stability of soil stabilized by cement alone.

## 4. Discussion of Mix Proportion

**Figure 14:**
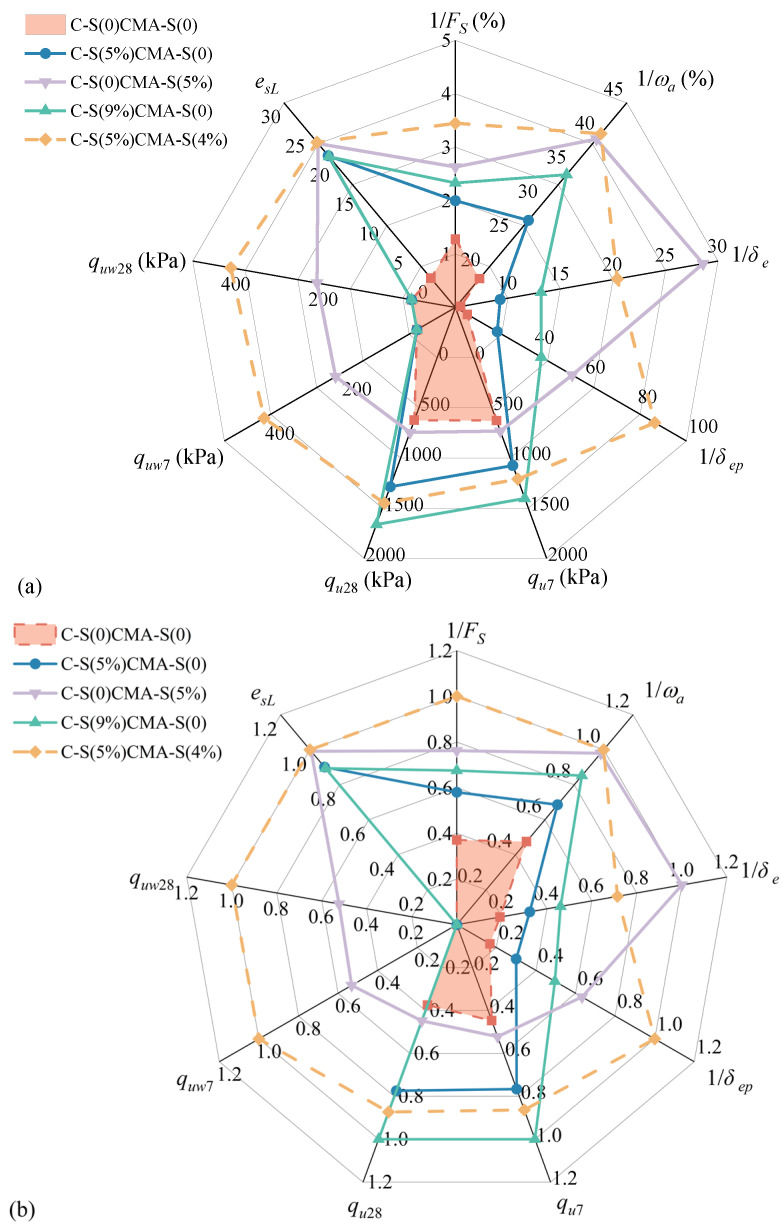
Engineering properties of the specimens varying with mix proportion: (**a**) actual values before normalization; (**b**) normalized values.

To compare the effects of composite and single stabilization, this study selected the untreated soil and four representative mixtures for systematic evaluation of free swelling ratio (*F_S_*), equilibrium moisture absorption (*w_a_*), unloaded swelling ratio (*δ_e_*), loaded swelling ratio (*δ_ep_*), linear shrinkage (*e_sL_*), and unconfined compressive strength before and after wet–dry cycles at 7 d and 28 d, totaling nine indicators. As shown in Figure 14, for swelling–shrinkage indicators, larger values indicate higher expansiveness; therefore, their reciprocals were used for evaluation, with larger values representing better performance. After processing, all indicators in Figure 14a follow the same criterion, where larger values indicate superior performance [55]. Figure 14a results show that when C-S is 5%, the 7-day compressive strength (*q*_u7_) increases by approximately 1.7 times, and the 28-day compressive strength (*q*_u28_) increases by about 2.1 times. However, cement alone has a limited effect on swelling–shrinkage, and the soil remains highly expansive. When C-S is increased to 9%, 1/*F_S_* is 2 and 1/*w_a_* is 27.78, still below the non-expansive soil criteria (1/*F_S_* > 2.5 and 1/*w_a_* > 40), and specimens subjected to dry–wet cycles may even collapse. In the composite stabilization group, adding 4% MAM-S to a mixture with 5% C-S results in 1/*F_S_* of 3.45 and 1/*w_a_* of 40.49, meeting the non-expansive soil criteria, indicating that MAM effectively mitigates expansiveness. MAM alone improves swelling behavior, but *q*_u7_ and *q*_u28_ increase only by ≈1.12 times, and post-wet–dry-cycle strength remains low (≈200 kPa). Each indicator in Figure 14a was normalized by dividing its value by the corresponding maximum value [56,57], resulting in normalized values ranging from 0 to 1, as shown in Figure 14b.

Based on the normalized data, the TOPSIS (Technique for Order Preference by Similarity to Ideal Solution) method was applied according to Equations (15)–(18) to quantitatively rank the comprehensive performance of different mix designs [58,59].

First, the normalized value of the *i*-th mix design under the j-th evaluation indicator is denoted as *r_ij_*. When considering weights, it is calculated according to Equation (15).

(15) $$v_{ij}=w_{j}r_{ij}$$

In the equation, $v_{ij}$ represents the weighted normalized value of the *i*th mix design under the *j*-th indicator, and $w_{j}$ denotes the weight of the indicator.

It should be noted that each performance indicator plays an important role in the engineering application of stabilized expansive soil, and their weights may vary under different engineering scenarios [60,61]. In this study, no weighting was assigned to the indicators. In practical applications, weights can be determined according to specific working conditions.

Next, according to Equations (16) and (17), the Euclidean distances are calculated between the *i*-th mix design and the scheme with all performance indicators at their optimal values, as well as the scheme with all indicators at their worst values.

(16) $$D_{i}^{+}=\sqrt{\sum_{j=1}^{n}{\left(v_{ij}-v_{j}^{+}\right)}^{2}}$$

(17) $$D_{i}^{-}=\sqrt{\sum_{j=1}^{n}{\left(v_{ij}-v_{j}^{-}\right)}^{2}}$$

Here, $v_{j}^{+}$ and $v_{j}^{-}$ denote the maximum and minimum weighted normalized values of the *j*-th indicator among all mix designs, respectively. $D_{i}^{+}$ represents the distance between the i-th mix design and the optimal scheme, where a smaller value indicates closer proximity to the ideal state. $D_{i}^{-}$ represents the distance between the i-th mix design and the worst scheme, where a larger value indicates greater deviation from the worst state. Finally, the relative closeness is calculated according to Equation (18).

(18) $$C_{i}=\frac{D_{i}^{-}}{D_{i}^{+}+D_{i}^{-}}$$

Here, $C_{i}$ represents the relative closeness of the i-th mix design, generally ranging from 0 to 1. A larger $C_{i}$ indicates a better mix design, allowing different mix designs to be ranked based on their $C_{i}$ values, as shown in Table 5.

Overall, cement alone enhances strength but retains high swelling–shrinkage and limited water stability, while MAM alone mitigates swelling–shrinkage but provides limited strength improvement. In contrast, composite stabilization improves both strength and water stability, making it advantageous for mix design selection. Therefore, cement–MAM composite stabilization is recommended for engineering applications involving expansive soil.

## 5. Conclusions

Comparative laboratory tests were conducted on medium-expansive soil stabilized with cement, montmorillonite adsorption modifier (MAM), and cement–MAM composite stabilization. The key findings based on cement–soil ratio (C-S) and MAM-soil ratio (MAM-S) are as follows:

(1) Cement has limited influence on maximum dry density (*ρ*_dmax_) and optimum moisture content (*w*_opt_), whereas MAM exhibits a more pronounced effect; 6% MAM increases *ρ*_dmax_ by ≈0.04 g/cm^3^ and decreases *w*_opt_ by ≈2%.
(2) C-S of 9% reduces the free swelling ratio (*F_S_*) by 35% and equilibrium moisture absorption (*w_a_*) by 2.3%; MAM is more effective, with 5% MAM reducing *F_S_* by 40% and *w_a_* by 2.7%, lowering the swelling potential grade to the non-expansive soil range.
(3) MAM has a markedly greater effect on controlling the swell–shrink behavior of specimens than cement. At C-S = 5%, cement reduces the unloaded swelling ratio (*δ_e_*) by 7.4% and the loaded swelling ratio (*δ_ep_*) by 3%; MAM-S at 5% decreases *δ_e_* by 14.7% and *δ_ep_* by 5%, producing larger reductions. Cement has a negligible impact on linear shrinkage (*e_sL_*), whereas MAM shows significant mitigation; when C-S = 0, increasing MAM-S by 4% reduces *e_sL_* by 1.12%. However, at higher C-S, MAM’s effect on *e_sL_* diminishes.
(4) Cement markedly enhances unconfined compressive strength (*q*_u_); at C-S = 9%, 7-day and 28-day *q*_u_ are 2.2–2.7 times those of untreated soil. MAM-S does not affect the strength increase due to C-S. However, relying solely on cement is insufficient to ensure good water stability of the specimens, necessitating the incorporation of MAM. Overall, composite stabilization simultaneously improves strength and water stability, making it a preferable option for mix proportion selection.

However, this study has several limitations. First, the analysis of the MAM mechanism is mainly based on macroscopic results and literature, lacking direct physicochemical characterization; future work should incorporate elemental and microstructural analyses to clarify the mechanism. Second, the normalized strength model has only been validated within the investigated parameter range, and its reliability requires further verification using independent compressive strength data. Third, future studies should examine microstructure, stress–strain behavior, and freeze–thaw durability, and conduct mix optimization to identify a balance among engineering performance, environmental impact, and cost. Finally, additional field tests are needed to further assess the engineering applicability of composite-stabilized expansive soil.

## Figures and Tables

**Table 1:** Index properties of the expansive soil.

Parameter	Value
Specific gravity	2.72
Maximum dry density (g·cm^−3^)	1.61
Optimum moisture content, OMC (%)	22.28
Liquidity index	−0.19
Liquid limit (%)	74.9
Plasticity index	46.9
Free swelling ratio (%)	78
Equilibrium moisture absorption (%)	5.2

**Table 2:** Test matrix.

Test Type	Stabilized Group	C-S (%)	MAM-S (%)	Curing Time (d)
Compaction Test	Cement	1, 3, 5, 7, 9	0	7 and 28 d
	MAM	0	2, 3, 4, 5, 6	
	Composite	5	2, 3, 4, 5, 6	
		1, 3, 5, 7, 9	5	
Free Swelling Ratio, equilibrium moisture absorption, Loaded Swelling Ratio, Unloaded Swelling Ratio, Shrinkage and Unconfined Compressive Strength Test	Cement	1, 3, 5, 7, 9	0	
	MAM	0	2, 3, 4, 5, 6	
	Composite	1, 3, 5, 7, 9	4, 5, 6	

Note: Specimens for the unconfined compressive strength test were cured for 28 d, while all other tests used a curing period of 7 d.

**Table 3:** Shrinkage characteristic indices of the specimens.

MAM-S (%)	C-S (%)					
	0%	1%	3%	5%	7%	9%
0	× (×)	× (×)	× (×)	4.77% (11.00%)	4.69% (10.80%)	4.81% (10.92%)
2	5.17% (11.67%)	N/A	N/A	N/A	N/A	N/A
3	4.74% (10.88%)	N/A	N/A	N/A	N/A	N/A
4	4.62% (10.12%)	4.47% (9.96%)	4.35% (9.30%)	4.30% (8.85%)	4.31% (8.87%)	4.40% (8.91%)
5	4.34% (9.54%)	4.30% (9.17%)	4.26% (8.85%)	4.11% (8.46%)	4.22% (8.51%)	4.08% (8.31%)
6	4.05% (8.96%)	4.00% (8.02%)	3.99% (7.87%)	3.93% (7.78%)	3.81% (7.59%)	3.88% (7.63%)

Note: The data are presented in the format of “linear shrinkage ratio (shrinkage limit)”; “×” indicates that the specimen disintegrated during dry–wet cycling; N/A indicates that the corresponding mix proportion was not investigated.

**Table 4:** Compressive strength of the specimens after dry–wet cycles.

MAM-S (%)	C-S (%)					
	0	1	3	5	7	9
0	× (×)	× (×)	× (×)	× (×)	× (×)	× (×)
2	× (×)	N/A	N/A	N/A	N/A	N/A
3	× (×)	N/A	N/A	N/A	N/A	N/A
4	× (×)	× (×)	348 (370)	397 (413)	389 (491)	455 (568)
5	211 (216)	275 (303)	357 (405)	431 (517)	474 (532)	522 (611)
6	215 (221)	289 (321)	365 (416)	452 (525)	594 (654)	659 (792)

Note: The data are presented in the format of “7-d strength (28-d strength)”; “×” indicates that the specimen disintegrated during saturation; N/A indicates that the corresponding mix proportion was not investigated.

**Table 5:** Ranking of mix design schemes.

Mix Proportion	$D_{i}^{+}$	$D_{i}^{-}$	$C_{i}$	RANK
C-S(0) MAM-S(0)	2.39	0	0	5
C-S(5%) MAM-S(0)	1.83	1.094	0.374	4
C-S(9%) MAM-S(0)	1.64	1.382	0.458	3
C-S(0) MAM-S(5%)	1.08	1.681	0.608	2
C-S(5%) MAM-S(4%)	0.34	2.249	0.868	1

## Data Availability

The original contributions presented in this study are included in the article. Further inquiries can be directed to the corresponding authors.
